# Water Extract of *Lysimachia christinae* Inhibits Trabecular Bone Loss and Fat Accumulation in Ovariectomized Mice

**DOI:** 10.3390/nu12071927

**Published:** 2020-06-29

**Authors:** Ki-Shuk Shim, Youn-Hwan Hwang, Seon-A Jang, Taesoo Kim, Hyunil Ha

**Affiliations:** 1 Herbal Medicine Research Division, Korea Institute of Oriental Medicine, Yuseong-daero 1672, Yuseong-gu, Daejeon 34054, Korea; angeloshim@kiom.re.kr (K.-S.S.); hyhhwang@kiom.re.kr (Y.-H.H.); white7068@kiom.re.kr (S.-A.J.); xotn91@kiom.re.kr (T.K.); 2 Korean Convergence Medicine Major KIOM, University of Science & Technology (UST), 1672 Yuseongdae-ro, Yuseong-gu, Daejeon 34054, Korea

**Keywords:** *Lysimachia christinae*, osteoporosis, ovariectomy, osteoclast differentiation

## Abstract

In Asia, extracts of *Lysimachia christinae* have been used for liver or urinogenital system-related diseases in traditional medicine. In this study, we investigated the effects of the water extract of *L. christinae* (WELC) on receptor activator of nuclear factor-kappa Β ligand (RANKL)-induced osteoclastic differentiation of bone marrow macrophages, and on osteoporosis and obesity in ovariectomy mice. RANK signaling pathways related to osteoclast differentiation were examined by real-time polymerase chain reaction (PCR) and western blot analysis. Additionally, we performed micro-computed tomography to assess trabecular bone loss, histological analysis for fat accumulation in adipose, liver, and bone tissues, and phytochemical profiling for WELC characterization. WELC significantly inhibited osteoclast differentiation by downregulating RANKL-induced mitogen-activated protein kinase (MAPK)/c-Fos/nuclear factor of activated T-cells (NFAT) signaling in osteoclast precursors and ovariectomy-induced trabecular loss by suppressing osteolcastic bone resorption. WELC markedly decreased ovariectomy-induced body weight gain and fat accumulation in adipose, liver, and bone tissues. Furthermore, ultra-high-performance liquid chromatography-tandem mass spectrometry (UHPLC–MS/MS) identified 16 phytochemicals in WELC when compared with the mass fragmentation of standard chemicals. Collectively, these results suggest that WELC might possess beneficial effects on postmenopausal osteoporosis by inhibiting osteoclast differentiation and obesity by suppressing fat accumulation.

## 1. Introduction

Menopause and estrogen decline induce significant physiological alterations in women’s health, including the development of osteoporosis and obesity. In the bone remodeling unit, estrogen participates in the regulation of bone resorption by osteoclasts and bone formation by osteoblasts [1,2]. Estrogen loss specifically weakens the induction of osteoclast apoptosis or decreases as the expression of osteoprotegerin, a decoy receptor of receptor activator of nuclear factor-kappa Β ligand (RANKL) decreasing osteoclast activity, on stromal cells, but increasing the expression of pro-inflammatory cytokines (tumor necrosis factor (TNF)-α, interleukin (IL)-1, and IL-6) to stimulate osteoclast resorption [3,4,5]. Thus, estrogen loss increases the bone resorption rate rather than the bone formation rate, resulting in net bone loss and osteoporosis. Furthermore, estrogen is involved in the lipolysis pathway or regulates enzyme expression in glucose and lipid metabolism [6,7]. Estrogen loss significantly increases abdominal fat distribution, lipid accumulation in the adipose tissue, and stimulation of adipocyte differentiation [8,9], possibly related to increased weight gain and metabolic diseases induced by menopausal obesity [10,11]. Estrogen therapy significantly attenuates weight gain, as well as the volume and number of adipocytes in the bone marrow, related to the retardation of bone remodeling and fracture repair [12,13,14], suggesting a correlation between obesity and osteoporosis in menopause. In postmenopausal women, estrogen therapy has been utilized as the initial therapeutic strategy to prevent osteoporosis or obesity; however, owing to the potential risk of developing cancer and other diseases, there exists a need for alternative therapeutic approaches [15,16]. Therefore, to manage or treat postmenopausal health concerning osteoporosis or obesity, exploring effective candidates from among nutraceuticals or pharmaceutical plants is gaining momentum in medical science [17,18].

*Lysimachia christinae*, a member of Primulacae family, has been used in traditional medicine to eliminate over-heating in the body and damp pathogen in the liver or urinogenital system [19]. Recently, various pharmacological activities of *L. christinae* on liver diseases have been investigated, including its potential to stimulate bile flow, treat cholecystitis, reduce cholesterol gallstones, and protect against acute alcohol-induced liver injury [20,21,22]. In addition, it activities on the urinary organ-related diseases by reducing urinary calculus, urinary tract infection, or kidney calcium oxalate crystal formation in animal and clinical study have also been reported [23,24]. Furthermore, phytochemical analysis has identified several flavonoids and phenolic components as bioactive constituents in *L. christinae*, including myricetin, kaempferol, and quercetin [25,26,27]. However, the effect of *L. christinae* on genital organ-related diseases by cessation of ovarian function including postmenopausal osteoporosis and obesity remains unexplored.

In the present study, we investigated whether the water extract of *L. christinae* Hance (WELC) could mitigate bone structural deterioration and obesity in ovariectomized (OVX) mice. We examined trabecular bone structural parameters by micro-computed tomography (micro-CT) analysis, as well as fat accumulation in the adipose tissue, liver, and bone marrow by histological analysis. Additionally, we examined the effect of WELC on RANK signaling in osteoclast precursors in vitro using bone marrow-derived macrophage cells (BMMs) and the resorption activity of mature osteoclasts on the bone mimetic surface. Using authentic marker components, ultra-high-performance liquid chromatography-tandem mass spectrometry (UHPLC–MS/MS) was utilized to characterize the phytochemical profile of WELC.

## 2. Materials and Methods

### 2.1. Materials

The phytochemical components (quinic acid, epigallocatechin, catechin, chlorogenic acid, epicatechin, schaftoside, isoschaftoside, quercitrin, rosmarinic acid, myricetin, phlorizin, quercetin, kaempferol, betaine, and p-coumaric acid) were purchased from Targetmol (Wellesley Hills, MA, USA). Neochlorogenic acid and (-)-gallocatechin were purchased from ChemFace (Wuhan, China). MS grade acetonitrile, water, and formic acid were obtained from Thermo Fisher Scientific (Rockford, IL, USA). *L. christinae* was obtained from the National Development Institute of Korean Medicine (Gyeongsan, Korea) and was extracted (0.5 kg) by refluxing with distilled water (3.5 L), concentrated under reduced pressure, and then dried using a vacuum freeze dryer. The WELC powder was store at −20 °C until use.

### 2.2. Osteoclast Culture and Bone Resorption Assay

To induce proliferation, BMMs were cultured in α-Minimum Essential Medium (MEM) (Thermo Fisher Scientific, Waltham, MA, USA) with 10% fetal bovine serum (Thermo Fisher Scientific), 1% penicillin/streptomycin (Thermo Fisher Scientific), and macrophage colony-stimulating factor (M-CSF; 60 ng/mL, R&D Systems Inc., Minneapolis, MN, USA). To induce osteoclast differentiation, BMMs pretreated with WELC for 3 h were cultured with RANKL (100 ng/mL) for 4 days. Multinucleated cells stained with tartrate-resistant acid phosphatase (TRAP) buffer (50 mM sodium tartrate and 0.12 M sodium acetate, pH 5.2) and Fast Red Violet (0.5 mg/mL; Sigma-Aldrich, St. Louis, MO, USA) were enumerated as osteoclasts. After culturing with WELC for 24 h, cell viability was assessed using Cell Counting Kit-8 (CCK-8; Dojindo Molecular Technologies, Rockville, MD, USA) to measure the amount of formazan dye generated by dehydrogenase in cells. For the bone resorption assay, mature osteoclasts, prepared as described previously [28], were cultured on Osteo Assay Surface plates (Corning, NY, USA), in the presence or absence of WELC for 16 h. After removing the cells using 5% sodium hypochlorite (Yuhan Co. Ltd, Seoul, Korea), the total resorbed area was measured using the ImageJ software (version 1.52h, National Institutes of Health, Bethesda, MD, USA).

### 2.3. Quantitative Real-Time Polymerase Chain Reaction (RT-PCR)

Total RNA was extracted from cells using the RNeasy Mini kit (Qiagen, Hilden, Germany) according to the manufacturer’s instructions. The same quantity of total RNA was reverse-transcribed to cDNA using TaqMan reverse transcription reagents (Applied Biosystems, Foster City, CA, USA). Quantitative RT-PCR was performed using the ABI QuantStudio 6 Flex RT-PCR system with the TaqMan Universal PCR Master Mix (Applied Biosystems). The primers used in this study were c-Fos (Mm00487425_m1), nuclear factor of activated T-cells, cytoplasmic 1 (NFATc1, Mm00479445_m1), dendrocyte expressed seven transmembrane protein (DC-STAMP, Mm01168058_m1), ATPase, H+ transporting, lysosomal 38kDa, V0 subunit d2 (Atp6v0d2, Mm00656638_m1), and glyceraldehyde 3-phosphate dehydrogenase (GAPDH, Mm99999915_g1) from the TaqMan Gene Expression Assay (Applied Biosystems). All reactions were run in triplicate. Relative expression of the target genes was calculated using the ΔΔCt method with GADPH gene as internal reaction control and expressed as fold change relative to the control untreated with RANKL and WELC. Experiments were repeated three times, and results from one representative experiment were shown.

### 2.4. Western Blot

Cells were lysed in radioimmunoprecipitation assay buffer containing protease and phosphatase inhibitor cocktail (Thermo Fisher Scientific, Waltham, MA, USA). The lysates were collected by centrifugation at 13,000× *g* for 30 min at 4 °C. Protein quantification was performed using the bicinchoninic acid protein assay kit (Pierce Biotechnology, Rockford, IL, USA). Equal quantities of total protein were separated using Mini-PROTEAN TGX™ Precast Protein Gels and transferred onto polyvinylidene fluoride membranes using Trans-Blot Turbo Transfer System (Bio-rad, Hercules, CA, USA). The membranes were blocked with 5% nonfat milk and incubated with primary antibodies against c-Fos, NFATc1 (Santa Cruz Biotechnology, Dallas, TX, USA), p-extracellular signal-regulated kinase (ERK), ERK, p-c-Jun N-terminal kinase (JNK), JNK, p-p38, p38, p-IκBα, IκBα, and β-actin (Cell Signaling Technology, Danvers, MA, USA). After incubation with horseradish peroxidase-conjugated secondary antibodies, specific bands were detected with a LAS-4000 image analyzer (Fujifilm, Tokyo, Japan) and Image Gauge software V3.0 (Fujifilm, Tokyo, Japan) using an enhanced chemiluminescence western blotting substrate (Bio-Rad, Hercules, CA, USA).

### 2.5. Animal Study

Animal experiment protocols were approved by the Institutional Animal Care and Use Committee (IACUC) of Knotus (Guri, Korea). Six-week-old female C57BL/6J mice (Japan SLC Inc., Shizuoka, Japan) were housed in a specific pathogen-free environment (22 °C ± 2 °C and 55 ± 5% humidity under a 12 h light/dark cycle). Following one-week acclimatization on a standard chow diet and water *ad libitum*, the mice were ovariectomized (OVX) or sham-operated. One week after surgery, the mice were randomly divided into four groups of six mice each: (1) Sham, (2) OVX, (3) OVX mice administered WELC 100 mg/kg/day (WELC-L), and (4) OVX administered WELC 300 mg/kg/day (WELC-H). The mice had free access to water and a normal-fat diet containing 10 kcal% fat (D12450B, Research Diets, New Brunswick, NJ, USA). WELC was administered by oral gavage once daily for five weeks. After 7 h of fasting, blood and tissue samples were obtained.

### 2.6. Measurement of Bone Turnover Markers

Serum levels of cross-linked C-telopeptide of type I collagen (CTX-I) and procollagen type I N-terminal propeptide (PINP) were measured using ELISA kits (Immunodiagnostic Systems Ltd., London, UK).

### 2.7. Micro-CT Analysis

Micro-CT analysis (SkySacn 1276, Bruker, Kontich, Belgium) was utilized to scan structural changes in the distal femur of mice. The scanned images were reconstructed using the SkyScan NRecon program and analyzed using SkyScan CTAn software (version 1.17.7.2, Bruker, Kontich, Belgium). The measured volume of the distal femur starting at 80 µm from the lower end of the growth plate and extending across 150 cross-sections (1.2 mm high). Bone morphometric parameters, including trabecular bone mineral density (BMD, g/cm^3^), trabecular bone volume fraction (BV/TV, %), trabecular number (Tb.N, mm), trabecular separation (Tb.Sp, mm), and trabecular thickness (Tb.Th, mm), were calculated.

### 2.8. Histological Analysis

The tissue samples were fixed using 10% neutral buffered formalin, dehydrated using a series of ethanol, embedded in paraffin, and sectioned to 5-µm thickness. Before dehydration, bone samples were decalcified in RDO Gold (RDO, Aurora, IL, USA). The sections were stained with hematoxylin and eosin, and adipocyte size and lipid accumulation were measured using the ImageJ software.

### 2.9. UHPLC-MS/MS Analysis

Samples were analyzed using a Dionex UltiMate 3000 system (Dionex Corp., Sunnyvale, CA, USA) equipped with a Thermo Q-Exactive mass spectrometer controlled by Xcalibur v.3.0 and Tracefinder v.3.2 software (Thermo Fisher Scientific, Bremen, Germany). Chromatographic separation was performed using a Dionex UltiMate 3000 system, equipped with a C18 column (Acquity BEH, 100 × 2.1 mm, 1.7 μm) at 35 °C. The mobile phase for UHPLC was a gradient system consisting of 0.1% formic acid in water (solvent A, v/v) and acetonitrile (solvent B), at a flow rate of 0.25 mL/min. A gradient program was as follows: 97% A, 0.0–1.0 min; 97–85% A, 1.0–2.0 min; 85–50% A, 2.0–13.0 min; 50–100% A, 13–20 min; 0–0% A, 20.0–23.0 min; 97–97% A, 23.5–27.5 min. The injection volume of the sample was 3 μL. The Q-Exactive mass spectrometer was operated in positive and negative ion switching modes with the following ionization parameters: spray voltage, 3.5 kV; capillary temperature, 350 °C; S-lens radio frequency level 50; sheath gas flow rate, 40 arbitrary units; auxiliary gas, 10 arbitrary units. Mass spectra were acquired in full MS and ddMS2 scan modes with the following parameters: ion scan range, 100–1500 m/z; resolution of MS scans, 70,000; resolution of MS/MS scans, 17,500; maximum injection time 100 ms; and normalized collision energy, 25 eV.

### 2.10. Statistical Analysis

For the in vitro study, data are presented as mean ± standard deviation, and as mean ± standard error of the mean for the in vivo study. Data were analyzed by a one-way analysis of variance (ANOVA) and Dunnett’s post hoc test or a two-way ANOVA and Bonferoni’s post hoc test using GraphPad Prism version 8 (GraphPad, CA, USA). *p* values less than 0.05 were considered statistically significant compared to the control.

## 3. Results and Discussion

### 3.1. WELC Inhibits RANK Signaling during Osteoclast Differentiation

**Figure 1:**
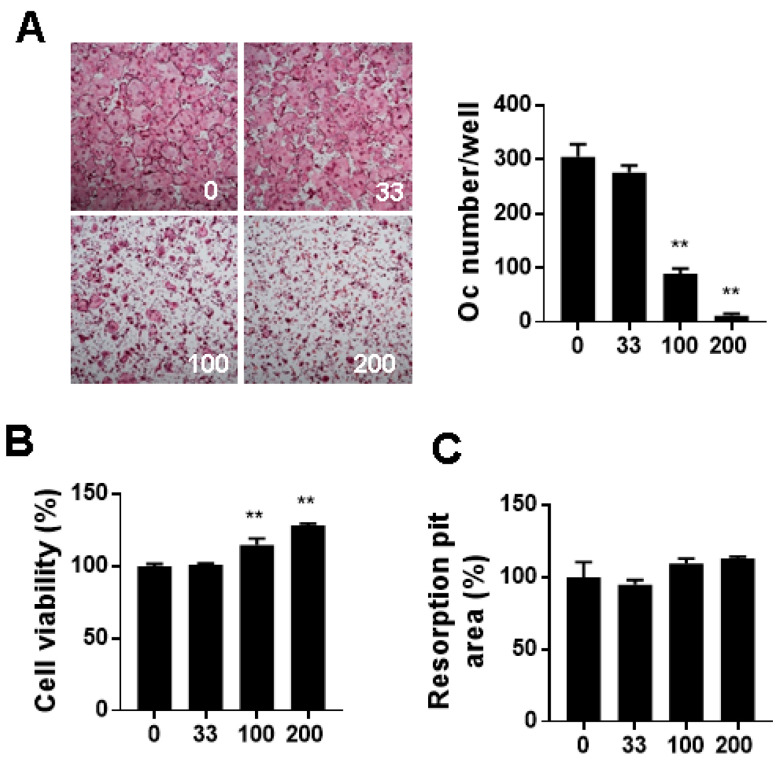
Inhibitory effects of WELC on osteoclast differentiation. (**A**) BMMs were cultured with WELC (33 µg/mL, 100 µg/mL, and 200 µg/mL) in the presence of RANKL for 4 days and then stained with TRAP staining solution. Representative images of TRAP-stained osteoclasts at 4× magnification. TRAP-stained multinucleated osteoclasts were enumerated. (**B**) BMMs were incubated with the indicated concentrations of WELC for 24 h followed by measurement of cell viability using the CCK-8 assay. (**C**) Mature osteoclasts were cultured with the indicated concentrations of WELC on the bone mimetic surface for 16 h to measure resorption pits. ** *p* < 0.01 versus vehicle control. BMMs, bone marrow-derived macrophage cells; TRAP, tartrate-resistant acid phosphatase; RANKL, receptor activator of nuclear factor-kappa Β ligand; WELC, water extract of *Lysimachia christinae*.

**Figure 2:**
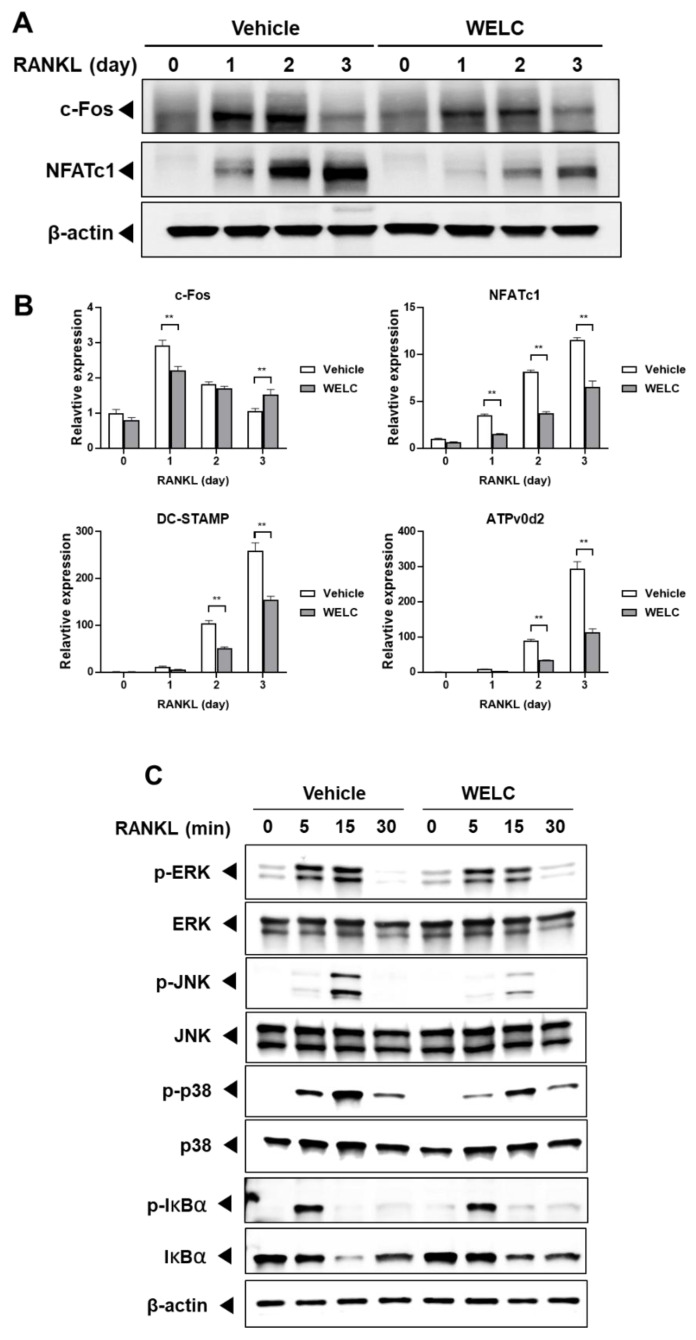
Inhibitory effects of WELC on RANK signaling pathways. (**A**,**B**) BMMs were pretreated with vehicle (distilled water) or WELC (200 µg/mL) and then simulated RANKL (50 ng/mL) for the indicated days. Day 0 represent BMMs untreated with RANKL for 1 day. (**A**) The protein levels of c-Fos, NFATc1, and β-actin were analyzed by Western blot analysis (**B**) The relative gene expression levels of c-Fos, NFATc1, DC-STAMP, and ATPv0d2 were analyzed by RT-PCR and expressed as fold change relative to each control (day 0 untreated with WELC). ** *p*  <  0.01 versus vehicle. (**C**) BMMs were pretreated with WELC for 3 h and then stimulated with RANKL for the indicated times. Protein levels were analyzed by western blot analysis with the indicated antibodies. BMMs, bone marrow-derived macrophage cells; RANKL, receptor activator of nuclear factor-kappa Β ligand; NFATc1, nuclear factor of activated T-cells cytoplasmic 1; DC-STAMP, dendrocyte expressed seven transmembrane protein; Atp6v0d2, ATPase, H+ transporting, lysosomal 38kDa, V0 subunit d2; WELC, water extract of *Lysimachia christinae*. RT-PCR, real-time polymerase chain reaction.

BMMs are precursor cells possessing the potential to differentiate into osteoclasts in the presence of RANKL. RANKL/RANK interaction activates RANK downstream signaling pathways, including mitogen-activated protein kinases (MAPKs) and NF-κB, to stimulate osteoclastogenesis [29]. First, we evaluated the effect of WELC on osteoclast differentiation of BMMs by counting the number of TRAP-stained multinucleated cells. WELC significantly suppressed RANKL-induced osteoclast formation (Figure 1A) but increased the viability of BMMs (Figure 1B). As mature osteoclasts demonstrate characteristics distinct from their precursors, we next examined whether WELC affects the bone resorption activity of mature osteoclasts. On the bone mimetic surface, WELC did not affect resorption pit formation by mature osteoclasts (Figure 1C), excluding the inhibitory activity of WELC during the late stage of osteoclast bone resorption. Based on these results, we investigated the effects of WELC on RANKL-induced signaling pathways to initiate osteoclast differentiation. c-Fos and NFATc1 are key transcription factors that are highly increased during RANKL-induced osteoclast differentiation [30,31]. We observed that WELC suppressed RANKL-induced mRNA and protein expression of both transcription factors (Figure 2A). Regarding WELC inhibition of osteoclast formation, as shown in Figure 1A, we further examined the expression of DC-STAMP and ATPv0d2 required for RANKL-induced osteoclast formation [32]. Consistent with WELC inhibition of osteoclast formation, WELC significantly suppressed RANKL-induced DC-STAMP and ATPv0d2 expression (Figure 2B). Moreover, we investigated WELC activity on RANKL-induced activation of MAPKs and NF-κB, which are early signaling pathways that induce c-Fos and NFATc1 expression [33,34,35,36]. WELC suppressed the activation of ERK, JNK, and p38, but not IκBα phosphorylation and degradation involving the classical NF-κB pathway (Figure 2C). Therefore, these results suggest that WELC specifically prevents the progress of osteoclast differentiation from precursor cells during the early stages of differentiation, by inhibiting RANKL-induced early signaling components and downstream transcription factors.

### 3.2. WELC Attenuates Bone Loss in OVX Mice

**Figure 3:**
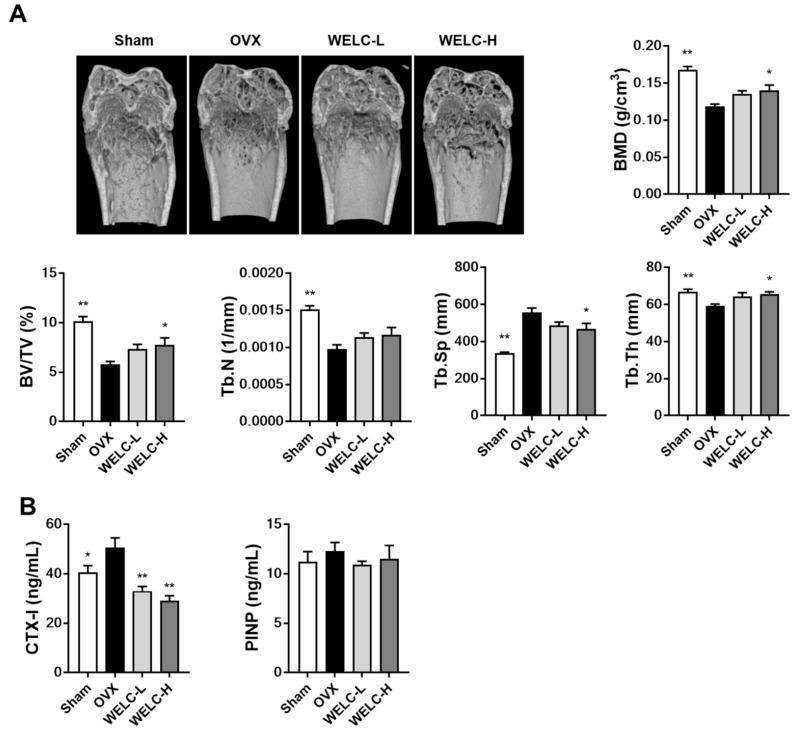
Inhibitory effects of WELC on OVX-induced bone loss. Sham or OVX mice were administered vehicle, WELC-L (100 mg/kg), or WELC-H (300 mg/kg) for five weeks. (**A**) The distal femora were scanned, and bone morphometric parameters (BMD, BV/TV, Tb.N, Tb.Sp, and Tb.Th) were analyzed. (**B**) Serum levels of CTX-I and PINP were measured. * *p * <  0.05, ** *p* < 0.01 versus OVX group. WELC, water extract of *Lysimachia christinae*; OVX, ovariectomized; BMD, bone mineral density; BV/TV, trabecular bone volume fraction; Tb.N, trabecular number; Tb.Sp, trabecular separation; Tb.Th, trabecular thickness; CTX-I, cross linked C-telopeptide of type I collagen; PINP, procollagen type I N-terminal propeptide.

The ovariectomy model is a standard model of postmenopausal osteoporosis exhibiting trabecular bone loss [37]. Ovariectomy increases osteoclast formation from precursors in the bone marrow and the number of osteoclasts per unit of bone surface that lead to increase in bone resorption and trabecular bone loss [38,39] To investigate whether WELC improves bone quality in OVX mice, we analyzed the trabeculae in the femur by micro-CT analysis. Micro-CT images showed that ovariectomy resulted in apparent changes in the trabecular bone structure of the distal femur in OVX mice (Figure 3A). We observed a compact trabecular structure in the sham, but OVX mice exhibited osteoporotic trabecular bone with less connectivity and thinner rod-like structures. Compared with the sham group, OVX mice exhibited significantly decreased BMD (29%), BV/TV (43%), Tb.N (35%), and Tb.Th (11%), demonstrating an increase in Tb.Sp (65%). A high concentration of WELC (WELC-H, 300 mg/kg) significantly improved OVX-induced bone deterioration by increasing BMD (18%), BV/TV (34%), and Tb.Th (11%), decreasing Tb.Sp (16%). In the animal model, BMD and BV/TV are positively correlated with trabecular bone strength and stiffness, while Tb.Sp is negatively correlated with the same; this predicts the correlation of these parameters with bone strength in the later life of animals [40]. Therefore, it could be suggested that the inhibitory activity of WELC on OVX-induced bone loss could protect against estrogen loss-induced osteoporosis and diminished bone strength observed during menopause.

To explore the mode of action of the bone-protective effect of WELC, we measured serum levels of CTX-I, a maker of bone resorption, and PINP, a maker of bone formation. In line with in vitro anti-osteoclastogenic activity, WELC markedly inhibited OVX-induced increase in CTX levels, while PINP levels were not altered in all groups (Figure 3B). Therefore, these results suggest that the protective effect of WELC on OVX-induced bone loss is attributed primarily to inhibition of osteoclast-mediated bone resorption.

### 3.3. WELC Inhibits Fat Accumulation in OVX Mice

**Figure 4:**
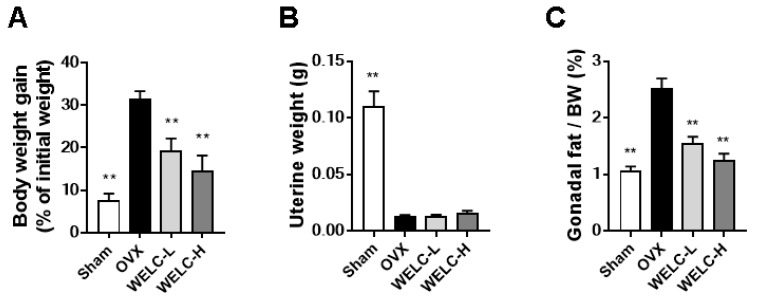

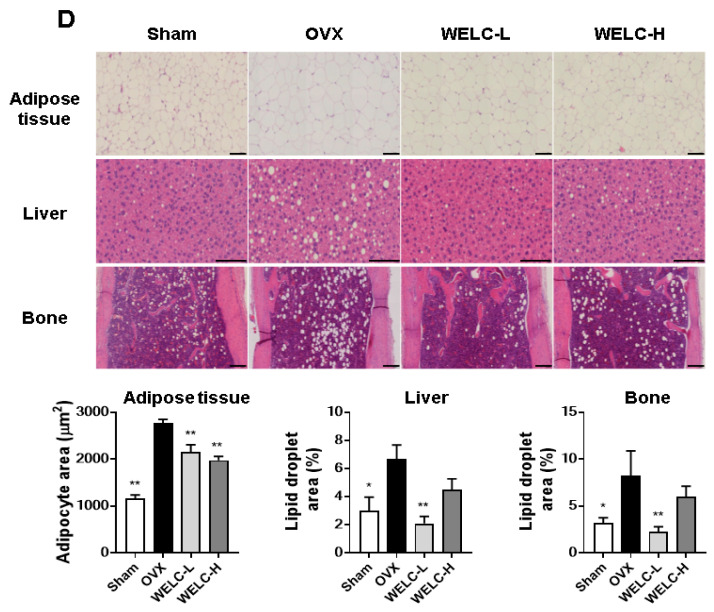
Inhibitory effects of WELC on OVX-induced fat accumulation. Sham or OVX mice were administered vehicle, WELC-L (100 mg/kg), or WELC-H (300 mg/kg) for five weeks. (**A**) Body weight gain during the experimental period, (**B**) uterine weight, and (**C**) gonadal fat weight were measured. (**D**) Histological analysis of adipose tissue, liver, and bone was performed using hematoxylin and eosin staining (scale bar, 100 µm). Image analysis of the adipocyte area or lipid droplets in each tissue was performed using the ImageJ program. * *p * <  0.05, ** *p* < 0.01 versus OVX group. WELC, water extract of *Lysimachia christinae*; OVX, ovariectomized.

In OVX rodent models, estrogen deficiency has been indicated as a major factor responsible for fat accumulation and fat distribution toward visceral adipose tissue [41,42]. As expected, OVX mice demonstrated an increase in body weight and gonadal fat, with a decrease in uterine weight, when compared with the sham group (Figure 4A–C). WELC-treated groups showed significantly reduced body weight and gonadal fat when compared with the OVX group. However, OVX-induced uterine atrophy was not ameliorated following WELC treatment, suggesting that WELC lacks estrogenic effects. Histological staining showed that WELC attenuated OVX-induced increased lipid accumulation in the liver and bone marrow, as well as reduced enlarged adipocytes in fat tissue (Figure 4D). These findings are consistent with a previous study demonstrating that *L. christinae* suppresses hepatic lipid accumulation and inflammatory infiltration induced by a high-fat and high-cholesterol diet in mice [21]. Furthermore, it has been reported that *L. christinae* protects against alcohol-induced liver injury in mice, possibly by inhibiting oxidative stress [22]. Thus, the antioxidant activities of WELC might contribute to its inhibitory action on fat accumulation. However, the underlying mechanisms need to be further investigated. Similar to the results of body weight gain, WELC at the high dose (WELC-H, 300 mg/kg) was more effective than the low dose (WELC-L) to decrease fat accumulation in adipose tissue. However, WELC-H was inefficient to decrease lipid accumulation in non-adipose tissues (liver and bone), whereas WELC-L was highly effective (Figure 4D). The mechanisms for this differential effect of WELC at the high dose on adipose and non-adipose tissues remain to be further studied.

### 3.4. Phytochemical Profiling of WELC

**Figure 5:**
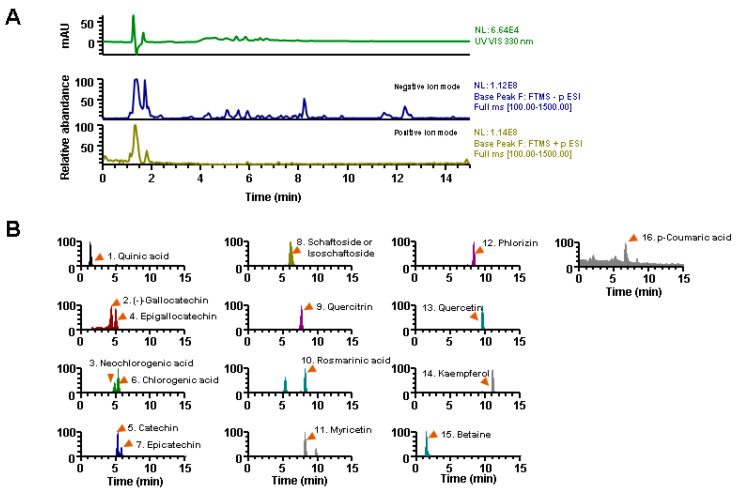
UHPLC-MS/MS analysis of WELC. (**A**) Ultraviolet and base peak chromatograms of WELC. (**B**) Extracted ion chromatograms of the identified components in WELC. UHPLC–MS/MS, ultra-high-performance liquid chromatography-tandem mass spectrometry; WELC, water extract of *Lysimachia christinae*.

Next, we investigated the phytochemical profile of WELC to determine the molecular basis of its pharmacological actions. UHPLC–MS/MS analysis identified diverse phytochemical components in WELC, including ten flavonoids (catechin, epigallocatechin, epicatechin, (-)-gallocatechin, kaempferol, myricetin, phlorizin, quercetin, quercitrin, and schaftoside), three polyphenols (chlorogenic acid, neochlorogenic acid, and rosmarinic acid), one cyclitol (quinic acid), one hydroxycinnamic acid (p-coumaric acid), and betaine (Table 1). The typical UV chromatograms at 254 nm and the extracted ion chromatograms for each component are shown in Figure 5. Reportedly, except for 3 components (quinic acid, schaftoside, and betaine), 13 individual components present inhibitory activities on osteoclast differentiation in vitro and OVX- or glucocorticoid-induced osteoporosis in animal models [43,44,45]. Additionally, some constituents possess anti-adipogenic activity by inhibiting the expression of key adipogenic transcription factors, decreasing the production of pro-inflammatory cytokines in 3T3-L1 cells, or by suppressing fat accumulation in animal models fed a high-fat diet [46,47]. Given that exposure of each WELC component on in vivo target tissue would be lower than the effective dose of individual components, WELC might demonstrate pharmacological efficacy in osteoporosis and obesity through additive and synergistic effects of these components present in WELC.

## 4. Conclusions

This study is the first to demonstrate the anti-osteoporotic and anti-osteoclastogenic effects of WELC. WELC inhibited bone loss by suppressing osteoclastic bone resorption in OVX mice and osteoclast differentiation in vitro by suppressing RANK signaling pathways inducing osteoclastogenic transcription factors. In addition, WELC suppressed ovariectomy-induced body weight gain and fat accumulation in adipose and non-adipose tissues. Furthermore, we identified 16 phytochemical components in WELC demonstrating inhibitory potential toward bone loss and fat accumulation. Taken together, these findings suggest that WELC is an attractive, natural therapeutic candidate for the prevention and treatment of postmenopausal osteoporosis and obesity.

## Figures and Tables

**Table 1:** List of identified components in WELC by UHPLC-MS/MS analysis.

No	R_t_ (Min)	Calculated (m/z)	Estimated (m/z)	Adducts	Error (ppm)	Formula	MS/MS Fragments (m/z)	Identifications
1	1.33	191.0561	191.0554	[M − H]^−^	−3.6119	C_7_H_12_O_6_	191.0553, 173.0081, 111.0073	Quinic acid
2	4.2	305.0667	305.0668	[M − H]^−^	0.5032	C_15_H_14_O_7_	305.0667, 179.0340, 125.0230	(-)-Gallocatechin
3	4.65	353.0878	353.088	[M − H]^−^	0.4012	C_16_H_18_O_9_	191.0552, 179.0339, 135.0437	Neochlorogenic acid
4	4.88	305.0667	305.0668	[M − H]^−^	0.5032	C_15_H_14_O_7_	305.0667, 179.0340, 125.0230	Epigallocatechin
5	5.11	289.0718	289.0719	[M − H]^−^	0.4823	C_15_H_14_O_6_	289.0716, 245.0816, 203.0709, 125.0230	Catechin
6	5.16	353.0878	353.088	[M − H]^−^	0.8415	C_16_H_18_O_9_	191.0553, 179.0340, 173.0445, 135.0439	Chlorogenic acid
7	5.64	289.0718	289.0719	[M − H]^−^	0.4823	C_15_H_14_O_6_	247.0246, 245.0816, 205.0501, 179.0340	Epicatechin
8	5.88	563.1406	563.1406	[M − H]^−^	−0.0089	C_26_H_28_O_14_	563.1415, 443.0985, 383.0767, 353.0662	Schaftoside or isoschaftoside
9	7.55	447.0933	447.0934	[M − H]^−^	0.3687	C_21_H_20_O_11_	284.0326	Quercitrin
10	8.03	359.0772	359.0775	[M − H]^−^	0.5949	C_18_H_16_O_8_	197.0447, 161.0232	Rosmarinic acid
11	8.04	317.0303	317.0304	[M − H]^−^	0.3328	C_15_H_10_O_8_	225.1116, 178.9975, 151.0023,	Myricetin
12	8.35	435.1297	435.1299	[M − H]^−^	0.5592	C_21_H_24_O_10_	435.1312, 273.0768, 209.0790, 152.9949	Phlorizin
13	9.58	301.0354	301.0355	[M − H]^−^	0.4732	C_15_H_10_O_7_	301.0352, 178.9977, 151.0025, 121.0282	Quercetin
14	11.02	285.0405	285.0406	[M − H]^−^	0.4504	C_15_H_10_O_6_	285.0406, 151.0029	Kaempferol
15	1.31	118.0863	118.0866	[M + H]^+^	2.5669	C_5_H_11_NO_2_	118.0864	Betaine
16	6.46	165.0546	165.0547	[M + H]^+^	0.6863	C_9_H_8_O_3_	147.0440, 84.9603	p-Coumaric acid

All data were compared with the retention time (R_t_) and MS spectral data of authentic standards. UHPLC–MS/MS, ultra-high-performance liquid chromatography-tandem mass spectrometry; WELC, water extract of *Lysimachia christinae*.
